# Academic publishing requires linguistically inclusive policies

**DOI:** 10.1098/rspb.2023.2840

**Published:** 2024-03-13

**Authors:** Henry Arenas-Castro, Violeta Berdejo-Espinola, Shawan Chowdhury, Argelia Rodríguez-Contreras, Aubrie R. M. James, Nussaïbah B. Raja, Emma M. Dunne, Sandro Bertolino, Nayara Braga Emidio, Chantelle M. Derez, Szymon M. Drobniak, Graham R. Fulton, L. Francisco Henao-Diaz, Avneet Kaur, Catherine J. S. Kim, Malgorzata Lagisz, Iliana Medina, Peter Mikula, Vikram P. Narayan, Christopher J. O'Bryan, Rachel Rui Ying Oh, Ekaterina Ovsyanikova, Katharina-Victoria Pérez-Hämmerle, Patrice Pottier, Jennifer Sarah Powers, Astrid J. Rodriguez-Acevedo, Andes Hamuraby Rozak, Pedro H. A. Sena, Nicola J. Sockhill, Anazélia M. Tedesco, Francisco Tiapa-Blanco, Jo-Szu Tsai, Jaramar Villarreal-Rosas, Susana M. Wadgymar, Masato Yamamichi, Tatsuya Amano

**Affiliations:** ^1^ School of the Environment, University of Queensland, Brisbane, Queensland, Australia; ^2^ Friedrich Schiller University Jena, Jena, Germany; ^3^ Department of Ecosystem Services, Helmholtz-Centre for Environmental Research – UFZ, Leipzig, Sachsen, Germany; ^4^ German Centre for Integrative Biodiversity Research (iDiv) Halle-Jena-Leipzig, Leipzig, Saxony, Germany; ^5^ Department of Architecture, Massachusetts Institute of Technology, Cambridge 02139-4307, USA; ^6^ GeoZentrum Nordbayern, Friedrich-Alexander-Universität Erlangen-Nürnberg, Erlangen, Bayern, Germany; ^7^ Department of Life Sciences and Systems Biology, University of Turin, Italy; ^8^ Institute for Molecular Bioscience, University of Queensland, Brisbane, Queensland, Australia; ^9^ School of Biological, Earth and Environmental Sciences, University of New South Wales, Sydney, New South Wales, Australia; ^10^ Institute of Environmental Sciences, Jgaiellonian University, Krakow, Poland; ^11^ Environmental and Conservation Sciences, Murdoch University, Murdoch, WA 6150, Australia; ^12^ The University of Chicago, Chicago, IL, USA; ^13^ School of BioSciences, University of Melbourne, Melbourne, Victoria, Australia; ^14^ Czech University of Life Sciences Prague, Praha, Praha 12844, Czech Republic; ^15^ Technical University of Munich, Munchen, Bayern, Germany; ^16^ University of Exeter, Exeter, UK; ^17^ Buck Institute for Research on Aging, Novato, CA, USA; ^18^ Moreton Bay Research Station, University of Queensland, Australia; ^19^ Department of Ecology, Evolution & Behavior, University of Minnesota, Minneapolis, MN 55108, USA; ^20^ Centre for Health Services Research, Faculty of Medicine, University of Queensland, Australia; ^21^ National Research and Innovation Agency Republic of Indonesia, Jakarta Pusat, DKI Jakarta, Indonesia; ^22^ Research Center for Plant Conservation, Botanic Gardens, and Forestry, National Research and Innovation Agency Republic of Indonesia, Bogor 16911, Indonesia; ^23^ Centro de Pesquisas Ambientais do Nordeste, Recife, Brazil; ^24^ School of Social Science, University of Queensland, Australia; ^25^ Department of Biological Resources, National Chiayi University, Chiayi City 600, Taiwan; ^26^ Australian Rivers Institute, Griffith University, Nathan, Australia; ^27^ Biology Department, Davidson College, Davidson, NC, USA; ^28^ Center for Frontier Research, National Institute of Genetics, Mishima, Shizuoka, Japan

**Keywords:** language barriers, academic publishing, inclusivity, biological sciences, society journals

## Abstract

Scientific knowledge is produced in multiple languages but is predominantly published in English. This practice creates a language barrier to generate and transfer scientific knowledge between communities with diverse linguistic backgrounds, hindering the ability of scholars and communities to address global challenges and achieve diversity and equity in science, technology, engineering and mathematics (STEM). To overcome those barriers, publishers and journals should provide a fair system that supports non-native English speakers and disseminates knowledge across the globe. We surveyed policies of 736 journals in biological sciences to assess their linguistic inclusivity, identify predictors of inclusivity, and propose actions to overcome language barriers in academic publishing. Our assessment revealed a grim landscape where most journals were making minimal efforts to overcome language barriers. The impact factor of journals was negatively associated with adopting a number of inclusive policies whereas ownership by a scientific society tended to have a positive association. Contrary to our expectations, the proportion of both open access articles and editors based in non-English speaking countries did not have a major positive association with the adoption of linguistically inclusive policies. We proposed a set of actions to overcome language barriers in academic publishing, including the renegotiation of power dynamics between publishers and editorial boards.

## 1. Introduction

Sharing scientific knowledge across the globe is key to addressing many global challenges and achieving Sustainable Development Goals (Target 17.6). Yet circulation of scientific knowledge remains geographically restricted. Monolingualism in academic publishing has created a language barrier for knowledge transfer, where scientific knowledge is produced in multiple languages but hegemonically pushed to be published in English. Consequently, scholars from countries where English is not widely spoken expend more cost and effort when publishing in English than scholars from countries where English dominates [1–4]. They also face the dilemma of achieving global visibility by publishing their work in English or making their work accessible to local communities by publishing in their native language. This tradeoff hinders the ability of scholars and communities to address both regional and global issues, such as the conservation of biodiversity [5]. It also hampers efforts to achieve diversity and equity in science, technology, engineering and mathematics (STEM).

Overcoming language barriers in STEM requires publishing policies that level the playing ground for scholars who are non-native English speakers and that facilitate the transfer of scientific knowledge between communities with diverse linguistic backgrounds [6]. As gatekeepers of scientific knowledge, academic publishers and journals are responsible for providing a fair system that supports non-native English speakers and disseminates knowledge across geographic and linguistic borders. However, individual publishers and journals have different values, incentives and resources to strive towards linguistic inclusivity. Here, we surveyed the practices and policies of 736 journals in biological sciences to assess their linguistic inclusivity, identify predictors of inclusivity, and highlight areas where publishers and journals can take action to increase diversity and equity in STEM. Below, we illustrate what those linguistically inclusive policies look like, noting that this constitutes a non-exhaustive list and additional linguistically inclusive policies will complement the efforts of journals to overcome language barriers in academic publishing.

### (a) Linguistically inclusive policies

Journals can support scholars who are not native English speakers in a variety of ways throughout all stages of the editorial process [6]. Here, we present some linguistically inclusive policies that journals in all fields of STEM should consider to support authors and readers from diverse linguistic backgrounds. (i) *Language of manuscripts*: publishing manuscripts and abstracts in other relevant languages would enhance the accessibility of scientific knowledge to communities in countries where English is not widely spoken. (ii) *Linguistic inclusivity statement*: a public statement declaring that manuscripts will be fairly assessed regardless of the perceived standard of English would signal the commitment of journals to overcome language barriers. (iii) *Language of guidelines*: providing author guidelines in multiple languages would assist authors in the preparation of their manuscripts and further signal that the journal values submissions from authors based in regions where English does not dominate. (iv) *Non-English-language references*: non-English-language literature can provide unique information, and encouraging authors to use this resource would enable comprehensive and globally relevant research, which is not possible when only citing English-language literature [7,8]. (v) *English editing services*: helping authors improve the readability of their manuscripts through English-language mentoring programmes or English editing services free of charge to authors would improve the editorial experience for authors, reviewers, and editors. (vi) *Linguistic instructions to reviewers and editors*: instructing both reviewers and editors to be aware of language biases and assess manuscripts based on their research attributes alone would contribute to a fairer assessment of manuscripts from authors who are non-native English speakers. Reviewers and editors should also be reminded that language norms vary among regions and that judging based only on what constitutes standard English might be exclusionary. (vii) *Machine translation tools*: implementing machine translation tools would improve the accessibility of published papers for non-native English-speakers [9].

## 2. Material and methods

### (a) Selection of journals and predictor variables

We examined the *2020 Journal Citation Reports* (JCR) from Clarivate Analytics and selected journals in biological sciences that publish content relevant to addressing the global biodiversity crisis. Those journals were listed under the following disciplines of the JCR: Biodiversity Conservation, Ecology, Entomology, Evolutionary Biology, Ornithology, Plant Sciences and Zoology. We also added to our sample six transdisciplinary journals that regularly publish articles in biological sciences: *Nature*, *Science*, *PNAS*, *PLOS Biology*, *Current Biology* and *eLife*. We excluded 26 out of 762 journals for any of the following reasons: (i) They did not publish content in English (*n* = 3), (ii) they were a book series (*n* = 3), (iii) their author guidelines were unavailable in their website (*n* = 3), (iv) their website was corrupted (*n* = 3), (v) they were out of the scope of their nominal discipline (*n* = 10), (vi) they were out of circulation (*n* = 2) or (vii) they only allowed submissions from members of a particular organization (*n* = 2).

To assess what predictors might have contributed to the adoption of linguistically inclusive policies, we collected the following information from the JCR for each journal: discipline, impact factor, percentage of open access articles, publication frequency (issues per year) and country of publication. We further searched on the journals' website for information about whether the journal was owned by a scientific society, whether the journal aimed at a global or regional readership, and whether the journal was published by either a for-profit or non-profit organization.

To test for the contribution of additional predictors related to the language spoken in the regions where either the journals were published or the editors were based, we used information from [10] to categorize countries as either English-speaking or non-English-speaking. For countries or territories where more than one language is commonly spoken, we only scored them as English-speaking if their most widely spoken language was English. We estimated the proportion of editors whose primary institution of affiliation was in countries where English is not the predominant language, non-English speaking countries hereafter, as a proxy of the linguistic diversity of editorial boards. Whereas we acknowledge that this metric does not necessarily reflect whether the editors are native English speakers or not, nor their English proficiency level, we expect that scholars working in regions where English is not predominantly spoken are well aware of the struggles associated with practicing science in languages other than English.

### (b) Data collection

We collected information about the linguistic policies of journals from both author guidelines and surveys to editors-in-chief between 2 September and 22 November 2021. Each co-author of this study collected information for a subset of journal titles following a data collection protocol. One author (HAC) compiled and cleaned the databases, cross-checking the information in instances where an observation was deemed odd, ambiguous or absent. We categorized the different policies and practices depending on what stage of the editorial process they affect (see electronic supplementary material, p. 6).

#### (i) Author guidelines

We examined the author guidelines of all journals in search of their linguistic policies and practices (electronic supplementary material, table S1). We also examined other sections of the journal websites to determine whether the published articles could be translated to other languages using machine translation tools. When a policy was not apparent in author guidelines, we explored other sections of the journal's website that looked like potential repositories of linguistic policies (e.g. ‘DE&I Statement’ and ‘English Language Editing’ from dropdown menus). For most journals, information was collected from the English version of their websites. However, some journals were assigned to co-authors that were originally from the region where the journal was published, making it possible to access author guidelines in languages other than English when available. We assumed that a particular policy or practice did not exist if it was not explicitly mentioned. The collected information does not account for linguistic policies that are described elsewhere (e.g. blog posts or the website of the societies that own the journal). When more than one option applied to a question, we only recorded the most inclusive one. For instance, if a journal both directed authors to commercial English editing services to improve the quality of English language and offered free English-language mentoring services to authors, we only recorded the latter.

#### (ii) Survey to editors-in-chief

We designed a survey to enquire editors about the linguistic policies and practices of journals, especially the policies for reviewers and editors that were not captured in the survey of the author guidelines (see the survey in electronic supplementary material, pp. 7–8). We examined the editorial board section of journals websites to identify the editors-in-chief and searched for their email address either on the same website or elsewhere (i.e. the website of their institution of affiliation). In instances where we could not retrieve this information, we searched for the contact details of an editor next in hierarchy (i.e. senior editor, specialty chief editor, handling editor) or, as a last resource, the managing editor or a representative of the editorial office. We emailed a standard message to all editors describing the aims of this study and asking them to complete the survey on behalf of the journal they represented. We also offered them the possibility to delegate this responsibility to other members of the editorial board or editorial office. We sent up to two reminders if the editors did not reply to the original message or complete the survey within two weeks.

### (c) Data analysis

We computed the answers to the different linguistic policy questions for each dataset separately. Some questions had two-level answers and others had three-level answers. For four questions, we collected answers from both author guidelines and surveys to editors-in-chief. To directly compare those paired results and assess whether the information posted in author guidelines matches the responses of editors-in-chief, we additionally computed the answers collected from author guidelines for the subset of journals whose editors-in-chief also responded to the survey.

We conducted regression analysis using four predictor variables: impact factor, the proportion of open access articles, the proportion of editors based in non-English speaking countries, and society ownership. We selected this set of variables because they display the lowest levels of correlation among themselves and, intuitively, they seem natural promoters or antagonists of linguistic inclusivity (electronic supplementary material, figure S1, and tables S2 and S3). To test whether the predictor variables were associated with the adoption of linguistically inclusive policies and practices, we conducted regression analysis for each question and dataset separately. For questions with two-level answers (binomial), we fitted a logistic regression model. For the guidelines dataset, we conducted the analysis in a mixed effect logistic regression framework to include the identity of the collaborator as a random effect and, thus, account for potential biases in data collection. For questions with three-level answers, we fitted an ordinal logistic regression model (see electronic supplementary material, p. 9, for more details). The curated datasets used in these analyses are archived on Zenodo (doi:10.5281/zenodo.10386753) [11].

## 3. Results and discussion

### (a) Panorama of linguistic policies

We collected information from the author guidelines of 736 journals that met our criteria. We also gathered responses from 262 editors-in-chief (36%) through our survey. Overall, the journals whose editors-in-chief completed the survey seemed to represent an unbiased sample of the total pool of journals considered in this study; the distribution of the values of most predictor variables was similar between the author guidelines dataset and survey to editors-in-chief dataset (electronic supplementary material, figure S2). However, editors-in-chief of the journals with the highest impact factor did not accept our invitation to complete the survey. Conversely, editors of society journals were more likely to complete the survey than editors of non-society journals ($\chi_{1}^{2}=15.02$, *p*-value = 0.0001).

**Figure 1.**
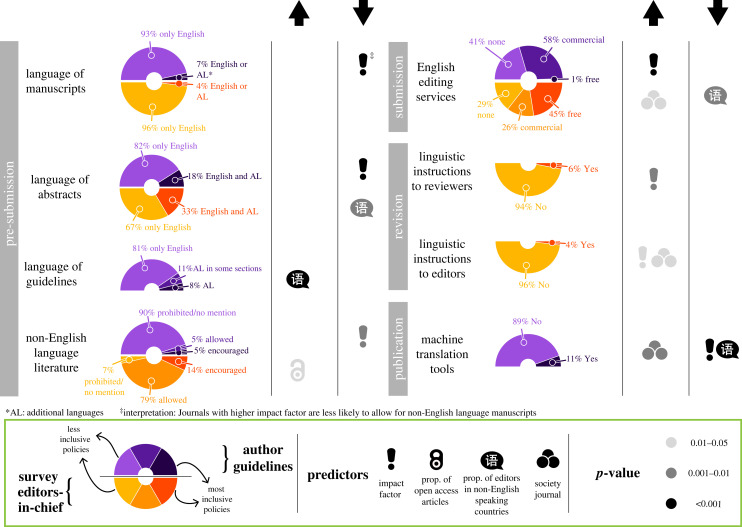
Panorama and drivers of linguistic inclusivity in academic publishing. Linguistic policies of journals as communicated in author guidelines (*n* = 736, the upper half of the donut) and answered in our survey by editors-in-chief (*n* = 262, the lower half) alongside the predictors that are associated either positively (upward arrow) or negatively (downward arrow) with the level of linguistic inclusiveness in policies.

Our assessment revealed that most journals are making minimal efforts to overcome language barriers in publishing (figure 1; electronic supplementary material, figure S3). As of late 2021, less than 7% of the journals allowed authors to publish articles in languages other than English. In the limited cases where journals allowed for publishing articles in additional languages, Spanish and French were the languages most frequently allowed. Publishing abstracts in an additional language was permitted by 33% of the journals according to editors-in-chief, but only 18% of all journals mentioned this possibility in their author guidelines. Less than 1% of journals (two out of 736) stated that manuscripts would not be rejected solely on the grounds of the perceived English standard and just 8% had their complete author guidelines accessible in at least one additional language, predominantly Spanish. Although most editors-in-chief indicated that they allow or encourage citing non-English-language literature, only 10% of the journals explicitly mentioned this in the author guidelines. Nearly half of the editors-in-chief claimed that journals offer free English-editing services, yet only 1% of journal guidelines offered information about in-depth assistance to authors through English-language mentoring programmes or professional editing services free of charge. Furthermore, only 6% and 4% of editors-in-chief reported that their journals instructed reviewers and editors, respectively, to avoid assessing manuscripts solely based on the perceived English quality. Only 11% of the journals implemented machine translation tools to read the online version of manuscripts in multiple languages (see electronic supplementary material, pp. 10–11, for a more detailed description of these results).

**Figure 2.**
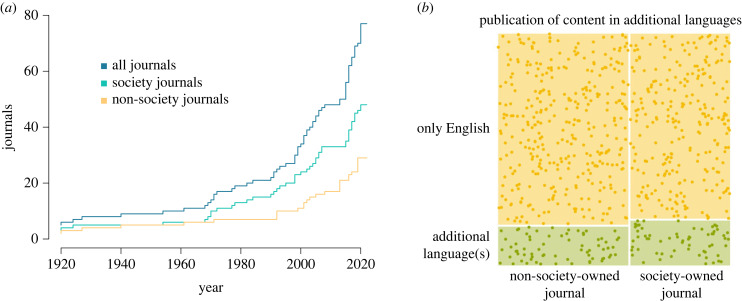
Patterns of publication of content in languages other than English between society and non-society journals. (*a*) Cumulative plot of the year in which journals started to publish content in languages other than English based on the answers by editors-in-chief to our survey (*n* = 77; 46 society journals and 31 non-society journals). Five journals adopted those policies before 1920. (*b*) Proportion of society and non-society journals that currently publish content in languages other than English as inferred from author guidelines (*n* = 736; 319 society journals and 417 non-society journals). The published content might correspond to abstracts, entire manuscripts or both.

Even though some journals have published content in more than one language since 1920 or earlier (figure 2), English hegemony has strengthened after World War II, being the first time in history where a single language dominates global scientific communication (see [12] and references therein). Prohibiting the publication of content in additional languages hinders the efforts to make STEM a multilingual enterprise again. Alternative policies aimed at alleviating the costs of the hegemony of English often fall short. For instance, although 58% of the journals directed authors to commercial English-language editing services in their guidelines, the cost of such services is prohibitive for many scholars. In lower-income countries, English-editing services cost might represent half of the average income of a PhD student [2]. Furthermore, while scholars who are not-native-English speakers from high-income countries tend to use professional English editing services for most of their papers, scholars from lower-income countries tend to not use any English editing services [1]. This can effectively reduce the publication rate, and overall participation in STEM, of scholars from lower-income countries.

Together, these results reflect that journals' support for non-native English-speaking authors and readers is limited and, quite often, author guidelines do not reflect the intentions of editors-in-chief to assist authors during the editorial process. The mismatch between the policies stated in author guidelines and the responses of editors-in-chief was particularly pronounced for policies related to citing non-English language literature and the provision of English editing services (electronic supplementary material, figure S3). Even though most editors-in-chief reported that their journals allow the citation of non-English-language literature, the policy is often not explicitly stated in author guidelines, which indirectly reinforces the tacit assumption that English is the *lingua franca* of STEM. We argue that journals should encourage authors to conduct broad-range searches to both maximize the scope of their work and value the scientific contributions of scholars who publish their work in languages other than English [8].

Three scenarios could explain the discrepancy between the English editing services that are offered in author guidelines and those that editors-in-chief declared: (i) author guidelines are not transparent or comprehensive, (ii) the information about these programmes is not advertised in author guidelines but elsewhere, or (iii) editors-in-chief’s perception of what constitutes free English editing support differ to ours. Our results lean support towards scenario (iii). While we only recorded a policy as free English editing support if it covered the expenses of editing services or provided in-depth language mentoring assistance, many editors-in-chief reported that editors suggest grammatical corrections or journals direct authors to English-language tutorials when given the possibility to describe the free English editing services that the journals offer.

### (b) Predictors of linguistic inclusivity

Impact factor, which is commonly perceived as a proxy of journal prestige, is non-randomly distributed among journals that differ in whether English is the primary language of the country they are based in and that have different geographic scopes in our dataset (electronic supplementary material, table S3). For instance, journals that were published in countries where English is not widely spoken or aimed at a regional readership tended to have a lower impact factor than journals published in English-speaking countries or aimed at a global readership. Moreover, non-English-language publications get fewer citations and, hence, a lower impact factor than English-language publications [13]. Because of the potentially disparate effects of these intertwined factors on inclusivity, impact factor can be associated both positively or negatively with the adoption of linguistically inclusive policies. On one hand, higher-impact-factor journals that aim at a global readership might enjoy of higher revenues to subsidize initiatives that assist authors from diverse linguistic backgrounds. On the other hand, lower-impact-factor journals that are primarily based in countries where English is not widely spoken might be more compelled to meet the needs of regional authors who are not native-English speakers.

Our results showed that higher-impact-factor journals were more likely to refer authors to commercial English-editing services in author guidelines than lower-impact-factor journals (figure 1; electronic supplementary material, table S4). This practice appears to be associated with a common commercial strategy of some publishers; higher-impact-factor journals tended to be published by for-profit publishers, which tend to advertise their own, or their commercial partners', English-editing services. Higher-impact-factor journals were also more likely to have reviewer and editor instructions about the importance of linguistically inclusive assessment of manuscripts. Furthermore, higher-impact-factor journals were less likely to publish manuscripts and abstracts in non-English languages and allow or encourage citing non-English-language references as per author guidelines. They were also less likely to implement machine translation tools on their websites. This indicates that higher-impact-factor journals implicitly target authors who can afford to pay for editing or translating services and aim at an English-proficient readership.

Open access publishing is generally perceived as a move towards inclusivity in STEM as it improves access to published scientific knowledge [14]. However, our findings reveal that open access is not necessarily a key predictor of linguistic inclusivity in journals (figure 1; electronic supplementary material, table S4). Open access had a significant association only with the citation of non-English-language literature; the editors-in-chief of journals with a higher proportion of open access articles were more likely to allow or encourage it. This finding, along with the fact that the cost of open access publishing represents a major barrier for scholars from lower-income countries [15], casts doubts on the contribution of the current open access models to reducing disparities in the global generation and dissemination of scientific knowledge.

The proportion of editors based in non-English-speaking countries may also be viewed as a driver of inclusivity in journals, since editors who have faced language barriers in their career may be more aware of the impacts of such barriers. Furthermore, journals with a higher proportion of editors based in non-English speaking countries tended to be published in countries where English is not widely spoken (electronic supplementary material, table S3). However, journals with a higher proportion of editors based in non-English speaking countries were less likely to publish abstracts in non-English languages and offer English editing services according to editors-in-chief. They were also less likely to implement machine translation on their websites (figure 1; electronic supplementary material, table S4). Only one instance of positive association with linguistic inclusivity was found; journals with a higher proportion of editors based in non-English speaking countries were more likely to provide author guidelines in multiple languages. This apparent paradox might reflect a lack of power of editorial boards to shape a journal's linguistic policies. For instance, in the field of economics, some major publishers, as opposed to each journal's editorial board, have set general English-language use policies for all their journals [16]. Alternatively, editors who have overcome language barriers might endorse established practices as a way to attain acceptance within a dominant scientific community and circumvent criticism from colleagues for promoting ‘disruptive’ policies [17].

Ownership by a scientific society was the clearest positive predictor of linguistic inclusivity in scientific publishing (figure 1; electronic supplementary material, table S4). Society journals were more likely to offer English-language editing services according to editors-in-chief (76.3% of society journals versus 64.2% of non-society journals offer at least one type of English editing service), instruct editors to assess the manuscripts regardless of the perceived English standard (5.8% of society journals versus 1.6% of non-society journals), and implement machine translation tools on their websites (15.7% of society journals versus 7.0% of non-society journals). Furthermore, society journals seem to have preceded, for several years, non-society journals in allowing the publication of non-English content (figure 2*a*). However, currently, both society and non-society journals publish non-English content in similar ratios ($\chi_{1}^{2}=0.6912$, *p*-value = 0.4058; figure 2*b*). As organizations with the capacity to define disciplinary norms and shape culture within academic communities [18,19], scientific societies are uniquely positioned to reform academic publishing towards linguistic inclusivity. Societies' greatest assets are their membership. Therefore, they have the responsibility to revise discriminatory practices and commit resources that support greater opportunities for members from historically marginalized groups, including scholars with limited English proficiency. Furthermore, membership can also be a driver of change towards inclusivity by establishing the foundation of English-mentoring programmes offered to potential authors of society journals [6].

### (c) A roadmap to overcome language barriers in academic publishing

To promote equitable participation of historically marginalized groups in STEM and maximize the generation and dissemination of scientific knowledge across the globe, academic publishing must undergo a cultural change [6]. Scientific societies demonstrably can play a critical role in fostering cultural shifts and we advocate to support community-led initiatives aimed at overcoming language barriers in STEM. For instance, since we collected the information examined in this study (November 2021), the Society for the Study of Evolution launched an English language mentoring programme to support authors upon submission to *Evolution* (April 2022) and the British Ecological Society integrated artificial intelligence (AI) proofreading tools to their journals’ submission system free of charge to authors (November 2022). Similarly, the Society for Open, Reliable, and Transparent Ecology and Evolutionary Biology started to accept manuscript submissions in Spanish and Portuguese in the *EcoEvoRxiv* preprint server (April 2023).

Beyond the adoption of the linguistically inclusive policies described in this study, we propose a set of actions that can further advance journals in this mission: (i) Journals should scrutinize and revise author guidelines to communicate their linguistic policies in a clear manner and reconcile author guidelines with the perception of editors [6,20]. (ii) The use of discriminatory language in author guidelines and arbitrary requests that disproportionally affect authors with limited or perceived limited English proficiency should be strongly discouraged [16]. Those exclusionary practices, such as requesting certificates of professional English-editing services, could impose a significant economic burden to scholars from lower-income countries. (iii) Authors should be allowed to harness AI tools, such as *ChatGPT* or *DeepL Write*, to proofread their manuscripts and submit both the original and the AI-proofread versions for the sake of transparency [21]. (iv) Scholars, editors and scientific societies should keep assessing the power dynamics between publishers and journal editorial boards in the development of linguistically inclusive policies and promote their renegotiation in the instances where it is deemed necessary. Finally, (v) journals should implement mandatory double-blind peer review systems to procure a fair assessment of manuscripts regardless of the English proficiency of the authors [4]. See a summary of these and additional recommendations in the electronic supplementary material (p. 12).

Overcoming language barriers in academic publishing is feasible and necessary, but it requires an understanding of the issues faced by scholars with limited English proficiency and a firm commitment from publishers, journals, and scientific societies to develop and implement linguistically inclusive policies. This is a pressing issue to address global challenges such as the biodiversity crisis [5], yet journals in the biological sciences currently fall short in their policies and practices to foster multilingual communities in STEM. Some of the recommendations that we have made here, and many more, have repeatedly been raised by different scholars (e.g. [1,2,4,6,8,9,16,21]), yet academic publishing practices do not seem to have experienced a substantial and generalized move towards linguistic inclusivity. We urge academic publishers and journals to revise their policies to identify any linguistic discrimination, educate themselves on scientific evidence related to language barriers [5–7] and the experience of their readers and potential authors [1,2], and commit resources to implementing linguistically inclusive policies.

## Data Availability

The curated datasets used in these analyses are archived on Zenodo: https://doi.org/10.5281/zenodo.10386753 [11]. Supplementary material is available online [22].
